# Primary central nervous system lymphoma in children: Insights from the three-year experience at the largest public-sector pediatric oncology center in Pakistan

**DOI:** 10.12669/pjms.41.13(PINS-NNOS).13443

**Published:** 2025-12

**Authors:** Rahat Ul Ain, Fiza Ismail, Laeeq Ur Rehman, Rabia Qaiser, Alia Ahmad, Mahwish Faizan

**Affiliations:** 1 Dr. Rahat Ul Ain, MBBS, FCPS, FCPS, FPNO. Department of Pediatric Hematology/Oncology, The Children’s Hospital Lahore, Pakistan; 2 Dr. Fiza Ismail, MBBS. Continental Medical College, Lahore, Pakistan; 3 Dr. Laeeq ur Rehman, MBBS, FCPS. Department of Pediatric Neurosurgery, The Children’s Hospital Lahore, Pakistan; 4 Dr. Rabia Qaiser, MBBS, FCPS. Department of Pediatric Radiology, The Children’s Hospital Lahore, Pakistan; 5 Dr. Alia Ahmad, MBBS, DCH, MRCPCH, FRCPCH, M.Sc. Department of Pediatric Hematology/Oncology, The Children’s Hospital Lahore, Pakistan; 6 Dr. Mahwish Faizan, MBBS, MCPS, FCPS, FPHO, M.Sc. Department of Pediatric Hematology/Oncology, The Children’s Hospital Lahore, Pakistan

**Keywords:** Brain Neoplasms, Developing countries, Lymphoma, Lymphoma primary central nervous system, Pediatrics

## Abstract

**Objective:**

Primary CNS lymphoma (PCNSL) in children is a rare disease, and this study aimed to document the experience of dealing with this in our geographical and resource settings.

**Methodology:**

It was an ambidirectional cohort study conducted at the Department of Pediatric Hematology/Oncology and the Department of Pediatric Neurosurgery at the University of Child Health Sciences, The Children’s Hospital Lahore, Pakistan from March 2024 to December 2024. This study included seven suspected and confirmed cases of PCNSL in children under the age of 16 years who presented to a public-sector specialized center in Pakistan over the three-year study period.

**Results:**

A total of seven cases of suspected PCNSL were included in the study. The median age at presentation was eight years, with a female-to-male ratio of 2.5:1. Most commonly presenting with a focal neurological deficit, with a median duration of symptoms of 12 weeks, and a median Lansky performance score of 50. Only 57% (04) of patients underwent surgical resection followed by adjuvant chemotherapy. Overall, 57% (03) of patients died, 29% (02) were lost to follow-up, and only 14% (01) are under treatment.

**Conclusion:**

PCNSL in children is equally rare in our part of the world, but has a dismal survival rate. Timely surgical intervention, improved supportive care, and a reduction in treatment abandonment might improve the prognosis.

## INTRODUCTION

Primary central nervous system lymphoma (PCNSL) is an uncommon Non-Hodgkin lymphoma (NHL) that arises from structures in the central nervous system (CNS) without any evidence of systemic disease.^1^ It accounts for only 3-5% of all CNS tumors and is rarer in children, with around 1%.^2^ The annual incidence of PCNSL is 0.4 per 100,000, with a 10-fold increase in incidence in the elderly and most commonly occurring between 50 and 70 years.^3^ The most common sites of involvement are the cerebral hemisphere, basal ganglia, corpus callosum, brainstem, and cerebellum, and the clinical manifestations depend upon the site of involvement.^4^ Over the past few years, the prognosis has improved significantly, mainly attributed to the use of high-dose methotrexate (MTX) chemotherapy, followed by consolidation and maintenance with radiotherapy and multi-agent chemotherapy, but relapses and treatment failures are common, with 5-year survival rates ranging from 30% to 50%.^4^ This is an aggressive disease, and the imaging characteristics might resemble various CNS tumors; a timely diagnosis and specific treatment are crucial for the best possible outcomes.^5^

PCNSL is rare in children; very few studies have been conducted in patients with PCNSL, mostly in the adult population, and from high-income countries.^6^-^9^ Understanding the disease manifestations, management, and survival rates in resource-limited settings can help improve the treatment protocols and outcomes in low/middle-income countries’ settings. The objective of this study was to demonstrate the clinical manifestations, management strategies, and experience with primary CNS lymphoma in resource-limited settings.

## METHODOLOGY

It was an ambidirectional cohort study conducted at the Department of Pediatric Hematology/Oncology and the Department of Pediatric Neurosurgery at the University of Child Health Sciences, The Children’s Hospital Lahore, Pakistan. After the approval from the institutional ethical committee (no. 803/CH-UHS, dated: March 5, 2024), the data were collected retrospectively from the hospital records since January 2022 and prospectively from March 2024 to December 2024. The cohort was followed until February 2025 for survival analysis. The sampling technique was non-probability, purposive sampling.

### Consent status:

Consent was taken from the guardian of the patients, and the patient identifiers were removed during data analysis.

### Inclusion criteria

- The study included all consecutive cases of children with confirmed and suspected cases of PCNSL under the age of 16 years.

### Exclusion criteria

- All suspected and diagnosed cases of CNS neoplasms other than PCNSL, relapsed cases, and patients more than 16 years of age were excluded from the study.

### Operational definitions

**Fig.1:**
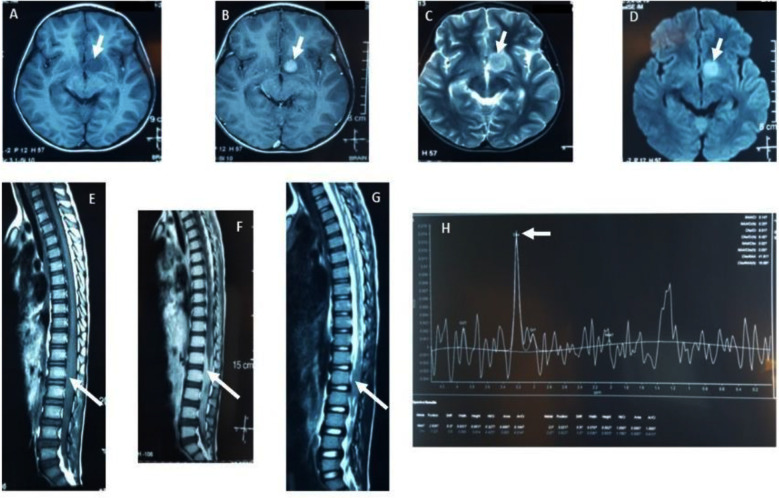
Neuroimaging of one of the patients with a primary left deep frontal lobe tumor and spinal metastasis (white arrows point out the lesions in the brain and the spinal cord). A: T1W Axial image, Brain: Hypo-intense lesion in left deep frontal lobe; B: T1W post-contrast axial image, Brain: Contrast-enhanced lesion in left deep frontal lobe; C: T2W Axial image, Brain; D: FLAIR Axial image, Brain; E: T1W sagittal image, whole spine: Diffuse soft tissue thickening around the terminal spinal cord in the spinal canal; F: T1W post contrast sagittal image, whole spine: Contrast enhancement in the terminal spinal cord; G: T2W image, sagittal image, whole spine: Hypo-intense lesion in the terminal spinal cord and H. MRS: Raised choline peak.

- A suspected case of PCNSL was defined as a case of primary CNS tumor with clinical and imaging findings suggestive of PCNSL after the multidisciplinary tumor board recommendation, but not confirmed histologically (Fig.1).
- A confirmed case of PCNSL was defined as a case of primary CNS tumor with histopathological findings suggestive of NHL.
- Event-free survival (EFS) was defined as the interval between the time of diagnosis till either the death of the patient or treatment abandonment.

### Data analysis technique:

The data on demographics, clinical findings, or outcomes were collected. The data were analyzed in terms of descriptive statistics, using mean, standard deviation, median, range, ratio, and percentages, due to the small number of cases.

## RESULTS

A total of seven cases of suspected and diagnosed PCNSL were registered during the three-year study period and included in the study. The total number of new CNS tumor cases during the study period was 549, with a proportion of 1.28% of suspected and diagnosed PCNSL cases. Four cases were diagnosed based on histopathology, while three were suspected based on radiology and could not undergo a surgical excision or biopsy to get a tissue diagnosis. The median and mean age was eight years (range: 4–13) (SD: 3.27), with a female-to-male ratio of 2.5:1. The most common presenting symptom was a focal neurological deficit, and the median duration of symptoms was 12 weeks (2-96 weeks). The median Lansky performance score at presentation was 50 (50 – 100). Clinically, cancer predisposition syndrome was suspected in only one case, while immune deficiency was suspected in none of the cases. Workup for immune deficiency or cancer predisposition syndromes was not done in any of the cases. Four patients underwent surgery, four received chemotherapy, and two abandoned treatment. The survival rate was only 14% (n=1) with a median event-free survival (EFS) of 12 weeks (2 – 24 weeks) (Table-I).

**Table-I:** Clinical characteristics of the study cohort with PCNSL.

Age / Sex	First Symptom	Symptom Duration	LPS	Primary Site	Histo-pathology	Metastasis/ CSF positive for disease	Chemo-Therapy	Outcome	EFS
***DIAGNOSED CASES (BASED ON HISTOPATHOLOGY)***									
4y / F	Left-sided hemiparesis	12 weeks	50	Right fronto-parietal region	High-grade B-cell NHL	None	UKCCSG group C	Treatment abandonment after COPADM 1	12 weeks
8y / F	Loss of vision	96 weeks	100	Right temporal region	Low-grade B-cell NHL (marginal zone lymphoma)	None	UKCCSG group C	On treatment	Alive
13y / M	Loss of vision	20 weeks	60	Right frontal lobe	Burkitt’s lymphoma	CSF positive for malignant cells	UKCCSG group C	Expired (TRM/SSI)	12 weeks
6y / M	Headaches and vomiting	2 weeks	50	Left deep frontal lobe + spinal cord mets (T11-L3)	High-grade B-cell NHL	CSF positive for malignant cells	UKCCSG group C	Expired (relapsed during maintenance)	22 weeks
***SUSPECTED CASES (BASED ON RADIOLOGY)***									
10y / F	Abnormal neck flexion	4 weeks	50	Midline corpus callosum	-	CSF not done	-	Expired (pre-op)	4 weeks
11y / F	Progressive paraplegia	2 weeks	70	D2- D6 intraspinal/ extramedullary	-	CSF not done	-	Expired (Pre-op)	18 weeks
4y / F	Headaches and vomiting	24 weeks	50	Left parieto-occipital lobe + corpus callosum	-	CSF not done	-	Treatment abandonment (Pre-op)	2 weeks

LPS: Lansky Performance Score, CSF: Cerebrospinal fluid, EFS: Event-free survival, TRM: Treatment-related mortality, SSI: Surgical-site infection, UKCCSG: UK Children’s Cancer Study Group, NHL: Non-Hodgkin Lymphoma, COPADM: Chemotherapy Regimen.

## DISCUSSION

PCNSL is an extremely rare type of tumor. More than 90 % of PCNSL are diffuse large cell B-cell Lymphomas (DLBCLs).^10^ In this study, the average age of patients was 8 ± 3.25 (4 – 13 years) years, with female predominance, which is contrary to the study by Attarbaschi et al., where the most common gender was male with a median age of 12.5 years. The most common manifestation of PCNL occurs as intracranial lesions (brain parenchyma)^11^ with frequent prevalence in the posterior fossa in the pediatric group.^12^ Similar to our study, where 71.42% (n=5) of patients presented with intracranial involvement. However, 28.57 % (n=2) had spinal involvement. Apart from that most commonly reported supratentorial region is the frontal lobe, and the cerebellum is seen to be affected in the infratentorial region.^11^ In this study, intracranial sites involved were: midline corpus callosum, right frontal-parietal, right parieto-occipital, right frontal lobe, and right temporal lobe.

The location PCNSL influences the clinical features observed in patients.^11^ Usually, intracranial involvement presents with symptoms of focal neurological deficit (70%) and raised intracranial pressure such as headache, vomiting 11 which is similar to this study where most common presenting complaint was focal neurological deficit 28.57% (n=2), headache and nausea/vomiting 28.57 % (n=2) (Table-I). The focal neurological deficit included symptoms such as lower limb weakness, walking difficulties, hemiparesis, and paraplegia. The literature suggests that the presentation of acute myelopathy in the pediatric group should be promptly evaluated with consideration of NHL in differential diagnosis.^13^ Other than that, presentation due to visual abnormalities in 15-20% of patients is reported in literature as compared to this study, where two patients presented with vision loss, 28.57% (n=2). Abnormal reflexes, which include exaggerated deep tendon reflexes and positive Babinski signs, were observed during examination in three cases (n=3). In a study by Attarbaschi et al., 44 % of patients presented with complaints of cranial nerve palsy^1^ compared to our study, where cranial nerve involvement (abducent nerve and facial nerve) was seen in two patients, 28.57% patients. In literature, cranial neuropathy (6 and 7) is seen as a feature of primary lipotomeningeal lymphoma.^11^

In this study, the average Lansky score was 61.42 ± 17.26. This contrasts with a study where the majority of patients had scored greater than 80%.^1^ Primary investigations usually done in PCNSL patients include brain and spine Magnetic Resonance Imaging (MRI), Cerebrospinal fluid (CSF) cytology, whole body Computed Tomography (CT), and viral markers.^12^ Contrast-enhanced MRI is preferred for cases of PCNSL, as 90% of cases present with contrast-enhancing lesions. The lesions are usually multiple and greater in size in immunocompetent patients as compared to immunocompromised patients.^12^ In this study, MRI findings include intra-axial multi-focal masses (n=2) and heterogeneously iso-to-hyperintense lesions on T2WI with irregular margins (n=1). Choline spikes were also observed on magnetic resonance spectroscopy (MRS). Biopsy and CSF analysis remain the modalities of choice to diagnose PCNSL.^14^ Biopsies were done in 71.14% (n=5) of the cases. Among these two cases were reported as high-grade B-cell NHL, one case of low-grade B-cell lymphoma n=1 and one case of Burkitt lymphoma n=1). The majority occurrence of B-cell lymphoma in this study aligns with the study by Benson et al., which states DLBCL is the most common type.^12^ Other investigations that were done include CSF cytology post-operatively and Pan CT. CSF cytology was positive for malignant cells in 28.57% of cases (n=2) as compared to the study by Song et al., in which malignant cells were detected in the CSF of 40% of patients.^11^ According to literature, a CSF analysis of multiple patients shows results as: Low glucose (54%), elevated protein (92%), and elevated White blood cell count (92%) with lymphocytic predominance and positive cytology.^11^ Metastasis in patients of PCSNL is rare; however, it contributes as a common cause of death in PCSNL patients.^14^ In this case series, Metastasis was reported in only one case (14.28%).

PCNSL is a highly chemosensitive and radiosensitive tumor.^12^ Due to the rarity of PCSNL and the unavailability of prospective studies, the decision regarding the choice of chemotherapy is difficult.^15^ The use of high-dose methotrexate, high-dose cytarabine, steroids, and no irradiation has been suggested for favorable outcomes and long-term disease control.^1^ In our study, a chemotherapy protocol, i.e., United Kingdom Children Cancer Study Group (UKCCSG) for High-grade B-cell NHL, is used in four patients. One out of four patients is under treatment, one patient had a relapse during treatment, one expired due to surgical-site infection and sepsis during treatment, and one was lost to follow-up after 1st session of chemotherapy. Overall, in this study, four patients (57%) have died, two patients (29%) have been lost to follow-up, and only one patient (14%) is currently under treatment. The Event-Free Survival (EFS) in this study was seen to be 12 weeks (2-24 weeks). Factors like age, sex, histopathology, and type of therapy have been reported to have little to no effect on Event-Free Survival (EFS) or Overall survival (OS), but in this study, a striking predilection is observed with the histopathology grade and EFS with the only survivor having a low-grade histopathology while all others (86%) with treatment failure had a high-grade histopathology. The presence of a pre-existing disorder significantly impacts the OS^1^, but none of the patients were diagnosed with any pre-existing disorder in this study cohort.

An improvement has been seen in prognosis in the last decade^14^, with a mean survival of 3-70 months (about six years) with the use of both chemotherapy and radiotherapy. However, radiotherapy is seen to be linked with early local failure and also associated with neuro-cognitive deficits.^15^

This study, however small, compares multiple aspects of PCSNL to existing literature, i.e., age and gender distribution, clinical presentation, investigation used, and treatment approach in a resource-limited setting, but larger cohorts with longer study periods are required for advanced statistical analyses and significant conclusions. Local literature from Pakistan in PCNSL is not available for comparison, but highlights the novelty of this study and the need for research in this field in our country.

### Limitations:

The small sample size and retrospective design of this study may introduce sampling or information bias. The long-term outcomes and overall survival could not be assessed in all the patients due to loss of follow-up. Furthermore, the study was conducted at a single tertiary care center, which may have limited applicability to other patient populations.

## CONCLUSION

PCNSL in children is uncommon and rare in our part of the world. Definitive diagnosis and timely surgical intervention were not done in a substantial proportion of patients. Female predominance and treatment abandonment were notable. The survival rates were dismal and were significantly associated with the grade of lymphoma. Immunodeficiency and cancer predisposition syndrome appeared to be infrequent. The association between surgical resection availability and survival highlights the need to strengthen neurosurgical support in pediatric neuro-oncology care. Larger study cohorts, longer study durations, and the availability of genetic testing for primary immune deficiency and cancer predisposition syndromes might better delineate the clinical characteristics of this group of patients in our population. An earlier diagnosis, timely surgical intervention, improved supportive care, and a reduction in treatment abandonment might improve the prognosis.

### Clinical recommendations:

Keeping in view the poor prognosis and acute presentations of PCSNL, it should be kept in the differential diagnosis to promptly start the definitive treatment, especially in children presenting with unexplained neurological deficits or visual disturbances. Prompt neuroimaging, preferably contrast-enhanced MRI, and timely surgical intervention play a crucial role in appropriate diagnosis and management. In diagnosed cases, high-dose methotrexate-based chemotherapy regimens, such as UKCCSG, can be effective. However, these regimens require close monitoring and improved supportive care. A national registry and multicenter collaboration are feasible yet significant steps to work on.

### Author`s Contribution:

**RUA:** Conceptualized the study, data analysis, manuscript writing. Final approval of the manuscript.

**FI:** Literature search, Data analysis and manuscript writing.

**LR and RQ:** Acquisition of data and drafting manuscript, Revising the content of manuscript.

**AA MF:** Interpretation of data and drafting manuscript. Revised the final version of manuscript.

All the authors have read and approved the final manuscript and are responsible and accountable for the accuracy and integrity of the work.
